# Beneficial rhizobacteria immobilized in nanofibers for potential application as soybean seed bioinoculants

**DOI:** 10.1371/journal.pone.0176930

**Published:** 2017-05-04

**Authors:** Priscilla Romina De Gregorio, Gabriela Michavila, Lenise Ricciardi Muller, Clarissa de Souza Borges, María Fernanda Pomares, Enilson Luiz Saccol de Sá, Claudio Pereira, Paula Andrea Vincent

**Affiliations:** 1 Instituto Superior de Investigaciones Biológicas (INSIBIO), CONICET-UNT and Instituto de Química Biológica “Dr. Bernabé Bloj”, Facultad de Bioquímica, Química y Farmacia, UNT. Chacabuco 461, San Miguel de Tucumán, Tucumán, Argentina; 2 Tecnano Produtos e Serviços Ltda, Rua Washington Luiz, 675, Porto Alegre, RS, Brazil; 3 Departamento de Solos, Faculdade de Agronomia, Universidade Federal do Rio Grande do Sul, Avenida Bento Gonçalves, 7712, Porto Alegre, RS, Brazil; ENEA, Italian Agency for New Technologies, Energy and Sustainable Economic Development, ITALY

## Abstract

Seed inoculation with plant growth promoting rhizobacteria (PGPR) is an ideal tool to supply the soil with a high density of beneficial microorganisms. However, maintaining viable microorganisms is a major problem during seed treatment and storage. In this work, an evaluation was made of the effect of bacterial immobilization in nanofibers on the stability (viability and maintenance of beneficial properties) of two potential PGPR, *Pantoea agglomerans* ISIB55 and *Burkholderia caribensis* ISIB40. Moreover, the impact of soybean seed coating with nanofiber-immobilized rhizobacteria on bacterial survival during seed storage and on germination and plant growth parameters was determined. Bacterial nanoimmobilization and subsequent seed coating with nanofiber-immobilized rhizobacteria were carried out by electrospinning. The results demonstrate that this technique successfully immobilized *P*. *agglomerans* ISIB55 and *B*. *caribensis* ISIB40 because it did not affect the viability or beneficial properties of either rhizobacteria. Seed coating with nanofiber-immobilized rhizobacteria improved *P*. *agglomerans* ISIB55 and *B*. *caribensis* ISIB40 survival on seeds stored for 30 days and contributed to the successful colonization of both bacteria on the plant root. Moreover, seed coating with *P*. *agglomerans* ISIB55 increased germination, length and dry weight of the root. Furthermore, seed coating with *B*. *caribensis* ISIB40 increased leaf number and dry weight of the shoot. Therefore, the technique applied in the present work to coat seeds with nanofiber-immobilized PGPR could be considered a promising eco-friendly approach to improve soybean production using a microbial inoculant.

## Introduction

Plant growth promoting rhizobacteria (PGPR) are beneficial soil bacteria that colonize roots and cause a positive effect on plants through direct and indirect mechanisms [1,2]. At present, PGPR inoculation constitutes a promising alternative strategy to reduce the use of chemical pesticides and fertilizers [3,4]. The beneficial characteristics of PGPR include: a) the capacity to increase plant nutrient uptake through phosphate solubilization [5], nitrogen fixation [6] and siderophore production [7]; b) the ability to produce phytohormones, such as indole-3-acetic acid (IAA) [8]; and c) biological control of pathogens, mainly caused by the synthesis of siderophores, antibiosis and induced resistance in crops against a broad spectrum of pests and diseases [9].

Soybean (*Glycine max* L.) is one of the oilseed crops with the highest production and consumption worldwide. It is widely used in the food industry for numerous products [10], while soybean oil is used for biodiesel production as an alternative for energy generation [11,12]. Nowadays, soybean constitutes a main export crop for several countries in South America, with the highest development in Argentina and Brazil [13,14]. Thus, the need arises for a better performance in soybean production based on the use of microbial inoculants that increase: a) rooting ability in the early stages, b) root surface for nutrient exchange between soil and plants and c) easy availability of essential nutrients such as nitrogen and phosphorus.

Inoculants can be applied to both seeds and soil. Nevertheless, because land application implies higher volumes of inoculums for suitable distribution of the bacteria, inoculation of seeds is the most effective and economic way of displaying PGPR in the rhizosphere. Thus, seed inoculation with PGPR is an ideal tool to deliver high densities of viable beneficial microorganisms to the soil where emerging plant roots may be colonized by them [15,16]. However, despite numerous laboratory studies that have demonstrated the ability of beneficial microorganisms to increase plant development, few bioinoculants adhered to seeds are commercially available because maintaining viable microorganisms is a major problem during seed treatment and storage [4,15,16].

Formulations with single and composite polymers have been studied as carriers of plant beneficial microorganisms in order to augment the efficacy and quality of bioinoculants and reduce costs and environmental impact [17–22]. Polymers are capable of encapsulating bacteria, protecting them from adverse environmental conditions and allowing their gradual release when these polymers are degraded by soil microorganisms. In addition, bacteria in polymers can be dry stored at room temperature for long periods [19]. Encapsulations in macro- and micro-alginate beads are the polymeric formulations of choice for the development of bioinoculants [23]. Moreover, these formulations have been suggested for seed treatment, considering the improved environmental persistence of bead-immobilized microorganisms [24]. However, the use of macro-alginate beads has two main disadvantages. The first drawback is that it requires additional seed treatment during sowing. This may be objected to because of inadequate agricultural education or the conservative cultural traditions of some small-scale growers wary of new technologies that could lead to non-inoculation of seeds. Moreover, farmers might be reluctant to incur extra costs and time. The second drawback is that when inoculant beads are mixed with seeds and sown, the beads can fall far from the seeds. After this, the bacteria released from the beads must migrate through the soil, facing competition from the native microorganisms. The use of micro-alginate beads could resolve these difficulties by employing seeds coated with “bead powder” at the handling facility, which are sold to the grower as “improved seeds” [23]. However, seed coating with microencapsulated PGPR requires additional adhesive substances such as lecithin and Resitol [19] and, being no easy task, it has, until now, only been conducted on an experimental scale [23]. Therefore, a simple and versatile method that directly allows seed coating with polymer-encapsulated bioinoculants could be a promising alternative.

Electrospinning is a technique in which a strong electric field is applied to a polymer solution which leads to physicochemical modifications resulting in nanoscale fibers [25]. The virtues of nanofibers, such as their small diameter, very large surface area, high porosity, easy fabrication and uniform morphology and composition, offer numerous advantages that have recently begun to be exploited in agriculture. Nanofibers are currently being used for seed treatment with chemical and biological substances that improve seedling establishment and control different infections by disease-causing organisms [26–28].

In our laboratory, *Pantoea agglomerans* ISIB55 and *Burkholderia caribensis* ISIB40 strains were isolated from different ecological niches (soybean and sugarcane rhizosphere, respectively) and selected as PGPR candidates for their potential beneficial properties (IAA production, phosphate solubilization, siderosphore synthesis and nitrogen fixation). It was hypothesized that the immobilization of PGPR by electrospinning preserves cell viability and intactness. Also, that soybean seed coating with nanofiber-immobilized PGPR: a) protects bacterial cells from adverse environmental conditions, ameliorating their survival during seed storage, b) improves seed germination, and c) contributes to root colonization by PGPR, which has a positive impact on plant growth. Thus, the aims of this study have been to: 1) evaluate the effect of bacterial immobilization in nanofiber on stability (viability and maintenance of beneficial properties) of both rhizobacteria, and 2) determine the impact of soybean seed coating with nanofiber-immobilized *P*. *agglomerans* ISIB55 or *B*. *caribensis* ISIB40 on rhizobacteria survival during seed storage, on germination and plant growth parameters.

## Materials and methods

### Microorganisms and culture conditions

*P*. *agglomerans* ISIB (Instituto Superior de Investigaciones Biológicas Culture Collection) 55 and *B*. *caribensis* ISIB40, isolated in Tucumán, Argentina, from soybean (*Glicine max* var. A8000 variety) and sugar cane rhizosphere (*Sacharis officinalis* var. LP 85–384 variety), respectively, were used in this study. The bacterial strains were kept in Brain Heart Infusion Broth (Merck, Germany) with 50% glycerol at -80°C.

Before experimental use, *P*. *agglomerans* ISIB55 and *B*. *caribensis* ISIB40 were grown in Yeast Mannitol Broth (YMB) [% (w/v): 1 mannitol, 0.05 K_2_HPO_4_, 0.02 MgSO_4,_ 0.01 NaCl and 0.05 yeast extract; pH 6.8; individual components obtained from Dinâmica Laboratories, Brazil], in a shaker at 28°C for 12 h and subcultured in the same medium at 28°C for 48 h. Viable bacteria were quantified by the successive dilution method, using saline as the dilution medium and Yeast Mannitol Agar (YMA) as the culture medium.

### Nanoimmobilization of rhizobacteria

The immobilization technique applied was electroespinning, in accordance with the methodology described by Damasceno *et al*. [27] with modifications. In order to carry out the immobilization test, 30% Polyvinyl alcohol (PVA) (Vetec™ reagent grade, 75% hydrolyzed, Mw 3000, crystalline; Sigma-Aldrich, Brazil) polymer solution was prepared. Polymer powder was dissolved in 10 ml of distilled water at 80°C until complete hydration of the polymer, which was autoclaved and stored at 4°C.

Cultures (pure or mixture with 5% glycerol) of *P*. *agglomerans* ISIB55 or *B*. *caribensis* ISIB40 were homogeneously mixed with 30% PVA polymer solution [1:1 (v/v) ratio]. The bacterial culture-polymer mix was subjected to electrospinning for 20 min from two syringes of 3 ml (BD, Belgium) connected to needles (TIP 22G x 1 in., BD, Belgium). A grounded collector was lined with aluminum foil to collect the spun fibers. Electrospinning parameters were set as follows: flow rate, 0.01mm·s^-1^; tip to collector distance, 12 cm; voltage, 12 kV or 22 kV.

### Nanofiber characterization

#### Viability of *P*. *agglomerans* ISIB55 and *B*. *caribensis* ISIB40

Cell viability was determined in the spinning solution (pre-immobilization) and in the nanofibers (post-immobilization) by the plate dilution method in YMA. The spinning solution was serially diluted, plated and incubated at 28°C for 24 h. The nanofibers were weighed, dissolved in saline at 25°C to release immobilized cells prior to serial dilution, and plated. Viability of *P*. *agglomerans* ISIB55 and *B*. *caribensis* ISIB40 in spinning solutions and nanofibers was determined as log_10_ colony forming units (CFU)·g^-1^. Survival rate during immobilization was expressed as NAI·NBI^-1^, where NAI and NBI are log_10_ CFU·g^-1^ after and before immobilization, respectively [29].

#### Studies of biochemical markers associated with plant growth promotion

For these studies, 10 mg nanofibers were cultivated in YMB supplemented with L-tryptophan 5 mM (IAA precursor) (Sigma-Aldrich, USA) at 28°C for 72 h. IAA production, phosphate solubilization, siderophore synthesis and nitrogen fixation were then studied, as described below. For the control, pure cultures of each microorganism were grown and evaluated under the same culture conditions. For each determination, two independent experiments were performed in triplicate.

IAA production was quantified by the colorimetric method described by Asghar *et al*. [30]. Bacterial cultures were centrifuged at 3000 *g* for 10 min. Supernatant (3 ml) was mixed with 2 ml Salkowski reagent (50 ml perchloric acid, 1.0 ml 0.5 M ferric chloride; individual components obtained from Dinâmica Laboratories, Brazil). Optical density (OD) at 530 nm was measured. The IAA concentration (μg·ml^-1^) was estimated based on the IAA standard curve (Sigma-Life Sciences, India).

Phosphate solubilization was determined, as previously described by Verma *et al*. [31]. Bacterial suspensions were adjusted to an OD_600nm_ = 0.2 and 20 μl were spotted on agarized medium supplemented with insoluble tricalcium phosphate and incubated at 28°C for 48 h. A clear halo formation around the colony indicated phosphate solubilizing capacity. Halo diameters and grown colonies were measured to obtain phosphate solubilization index (PSI), which is the ratio of the halo diameter to the colony diameter.

Siderophore synthesis was determined using the universal CAS assay described by Tortora *et al*. [32]. Bacterial suspensions were adjusted to OD_600nm_ = 0.2 and 20 μl were spotted on the surface of CAS-blue agar medium and incubated at 28°C for 48 h. A change in color from blue to orange was observed around the siderophore producing colonies. The diameter of the orange halo was determined by subtracting the colony diameter from the total diameter.

For determination of nitrogen fixation, the bacterial suspensions were washed with saline to remove nitrogen from the culture medium. The suspensions were then inoculated into tubes containing semisolid NFb medium [33]. The tubes were incubated at 28°C for 7 days and bacterial growth which induced color change in the medium was observed as qualitative evidence of atmospheric nitrogen fixation. As positive and negative controls, *Azospirillum brasilense* Az39 and *Escherichia coli* BW25113 were used, respectively.

### Seed coating with nanofiber-immobilized rhizobacteria

Soybean seeds [*Glycine* max L.; varieties, TEC 5936 IPRO and BRASMAX PONTA IPRO (RSF 7166 IPRO)] were surface-sterilized with 70% alcohol for 30 s followed by immersion in a solution of 4.5% sodium hypochlorite for 30 s and finally by three successive rinses in sterile distilled water. The seeds were dried on a sterile polypropylene tray for 30 min.

Fifty seed groups were placed in Petri dishes containing aluminum foil in the base. The dishes were then positioned above a vortex on a fixed base and subjected to electrospinning with the *P*. *agglomerans* ISIB55 or *B*. *caribensis* ISIB40 culture (with 5% glycerol)-polymer mix for 10 min. The electrospinning parameters were set as follows: flow rate, 0.01mm·s^-1^; tip to collector distance, 12 cm; and voltage, 22 kV. Following this, the seeds coated with nanofiber-immobilized rhizobacteria were stored in Petri dishes at room temperature under dark conditions. In order to evaluate the bacterial survival of the strains under study, sterile conditions were used during the seed storage time.

S1 Fig shows a diagram summarizing the process of soybean seed coating with nanofiber-immobilized rhizobacteria.

### Characterization of soybean seeds coated with nanofiber-immobilized rhizobacteria

The characterization of the seeds coated with nanofiber-immobilized *P*. *agglomerans* ISIB55 or *B*. *caribensis* ISIB40 (ISIB55-PVA or ISIB40-PVA-treated seeds) was evaluated, employing appropriate control seed treatments. The seeds were treated with the different components used during seed coating (YMB medium, glycerol, bacterial culture and PVA). Thus, the following control seed treatments were used a) ISIB55 or ISIB40-treated seeds: inoculated with *P*. *agglomerans* ISIB55 or *B*. *caribensis* ISIB40 cultures in YMB (with 5% glycerol), as described below; b) YMB-PVA-treated seeds: subjected to electrospinning with the YMB (with 5% glycerol)-polymer mix; c) YMB-treated seeds: inoculated with YMB with 5% glycerol; and d) untreated seeds. Prior to each treatment, the seeds were sterilized, as described above.

The ISIB55 or ISIB40-treated seeds were prepared by inoculating 50 seeds with 500 μl *P*. *agglomerans* ISIB55 or *B*. *caribensis* ISIB40 cultures containing 10^8^ CFU. The seeds were then placed on a polypropylene tray for 2 h for tegument drying and subsequently stored at room temperature under dark conditions.

#### Rhizobacteria survival in soybean seeds

Survival of *P*. *agglomerans* ISIB55 and *B*. *caribensis* ISIB40 was evaluated in a) ISIB55-PVA or ISIB40-PVA-treated seeds and b) ISIB55 or ISIB40-treated seeds. Bacterial recovery was determined immediately after polymer coating/inoculation and after 5, 10, 15, 20 and 30 days post-immobilization/inoculation.

For bacterial survival evaluation, ten treated seeds were placed in 10 ml saline and kept in a shaker for 20 min to detach the cells from the seeds. The viable cell number was then determined by the successive dilution method, using saline as the dilution medium and YMA as the culture medium. The viability of *P*. *agglomerans* ISIB55 and *B*. *caribensis* ISIB40 was determined as log_10_ CFU·seed^-1^.

#### Soybean seed germination

Seed germination and vigor tests were carried out in soybean seeds in accordance with the method described by Zhou *et al*. [34] with modifications. The experiments were carried out using a completely randomized design with 14 soybean seeds in Petri dishes for each treatment, with four replications per treatment. Two independent experiments were performed. The seeds were placed on sterile filter paper in Petri dishes and 4 ml sterile distilled water was added to the dishes. These were then incubated at 28°C and 1 ml of sterile distilled water was added to the dishes every 24 h. Over the next 4 days, the germinated seeds were counted and the root and shoot length was determined.

The evaluated germination traits included [35] [35] [36]:

$$Germination\;index=\sum \frac{number\;of\;germinated\;seeds\;on\;day\;N}{N\;days\;of\;seed\;germination}$$

$$Germination\;rate\;(Gr)=\left(\frac{number\;of\;germinated\;seeds}{number\;of\;seeds\;used\;in\;the\;assay}\right)\times 100$$

Vigorindex=(meanrootlength+meanshootlength)×Gr

#### Growth promotion study

These studies were performed in the glasshouse of the Soil Microbiology Laboratory in the Faculty of Agronomy of UFRGS (Federal University of Rio Grande do Sul). Two independent experiments were conducted using a completely randomized design with five polypropylene cups for each seed treatment.

Five hundred grams of sterile substrate composed of sand and vermiculite in a ratio of 2:1 were placed in the cups. In each cup, three soybean seeds were seeded at a distance of 1.5 cm from each other and from the cup edges. Finally, 100 ml Nutritive Solution [% (w/v): 13.6 KH_2_PO_4_, 24.6 MgSO_4_ 7H_2_O, 11.1 CaCl_2_, 7.5 KCl, 8 NH_4_NO_3_, 0.3 H_3_BO_3_, 0.01 ZnCl_2_, 0.004 CuSO_4_ 5H_2_O and 0.002 Na_2_Mo_4_; 1M FeEDTA solution; pH 6; individual components obtained from Dinâmica Laboratories, Brazil] diluted fourfold was added to each cup, with reposition every four days. On the 7th day, two out of three soybean seedlings from each cup were removed in order to preserve the one that showed the best growth.

The plants were harvested at the end of 25 days to record biometric observations. Leaf number, length of shoot and main root and dry biomass were evaluated to determine the effect of treatments on the growth parameters. The shoot and root of the plants were separated and dry weight was determined after drying in an oven at 70°C for 48 h.

### Scanning electron microscopy

Nanofibers, seed husks and soybean plant roots were assessed using scanning electron microscopy (SEM). The samples were affixed to sample stubs and subsequently coated with gold. Micrographs were then obtained using JEOL JSM 6060 equipment (Oberkochen, Germany) at an acceleration voltage of 10 kV and a sample to detector distance of 5 mm. Nanofiber mean diameter was calculated by measuring these in different points across three SEM images. Data were expressed as mean diameter ± standard error.

In order to obtain root samples, seeds from each treatment (described above for characterization studies) were placed in test tubes (length 250 mm; diameter 24 mm) containing 20 ml sterile Nutritive Solution with 0.6% agar (Dinâmica Laboratories, Brazil). The tubes were then maintained at 28°C for 48 h under dark conditions and subsequently placed in a chandelier with a photoperiod of 12 h of daylight for 7 days. The roots were cut, rinsed in distilled water and fixed in 3% glutaraldehyde (Vetec Laboratories, Brazil) in 0.1 M sodium phosphate buffer [% (w/v): 1.38 NaH_2_PO_4_.H_2_O, 2.68 Na_2_HPO_4_.7H_2_O; pH 7.0; individual components were obtained from Ecibra Laboratories, Brazil]. The tissue was washed three times with distilled water and 0.1 M sodium phosphate buffer (1:1 proportion) for 30 min and then dehydrated through a series of increasing acetone concentrations: 30% for 10 min, 50% for 10 min, 70% for 10 min, 90% twice for 10 and 20 min and 100% acetone twice for 10 and 20 min. Following this, critical point drying (BALZERS CPD030) was carried out.

### Statistical analysis

Analysis of variance (ANOVA) using a general linear model was applied to determine the effects of the factors evaluated in each of the following processes: a) bacterial nanoimmobilization: time of the immobilization process (pre and post-immobilization), bacterial culture (with or without glycerol) and immobilization conditions (voltage, 12 or 22 kV); and b) *P*. *agglomerans* ISIB55 or *B*. *caribensis* ISIB40 survival in soybean seeds: seed treatments (ISIB55-PVA or ISIB40-PVA-treated seeds and ISIB55 or ISIB40-treated seeds), seed varieties (TEC 5936 IPRO and RSF 7166 IPRO) and storage time (0, 5, 10, 15, 20 and 30 days post-treatment). The number of *P*. *agglomerans* ISIB55 and *B*. *caribensis* ISIB40 viable cells (log_10_ CFU·g^-1^ or log_10_ CFU·seed^-1^) was the response of interest analyzed in the different processes.

ANOVA, using a general linear model, was also applied to analyze: a) the effects of the condition in which *P*. *agglomerans* ISIB55 and *B*. *caribensis* ISIB40 were inoculated (cells in nanofibers or bacterial cultures) on IAA production, phosphate solubilization and siderophore synthesis; and b) the effects of seed treatment (ISIB55-PVA, ISIB40-PVA or YMB-PVA-treated seeds and ISIB55, ISIB40 or YMB-treated seeds) on seed germination and plant growth parameters.

In each analysis, significant differences (*P* < 0.05) between mean values were determined by Tukey’s test using InfoStat statistical software.

## Results

### Immobilization of beneficial rhizobacteria in nanofibers

One of the aims of this work was the immobilization of two beneficial isolates (*P*. *agglomerans* ISIB55 and *B*. *caribensis* ISIB40) in polymers by electrospinning in order to evaluate the effect of this treatment on the efficacy and quality of these bacteria as inoculants.

Firstly, the survival of *P*. *agglomerans* ISIB55 and *B*. *caribensis* ISIB40 was evaluated to determine if the immobilization process, under different conditions (bacterial cultures, pure or mixture with 5% glycerol; and voltage, 12 or 22 kV), was capable of modifying bacterial viability. The electrospinning conditions were selected on the basis of the results of Salalha *et al*. [37], who determined that glycerol addition conferred a protective effect on the viability of microorganisms against immobilization-induced stress. On the other hand, voltages of 12 and 22 kV were used as they were found to allow for appropriate immobilization of the different microorganisms [27,37,38].

The recovered CFU·g^-1^ from the nanofibers for both *P*. *agglomerans* ISIB55 and *B*. *caribensis* ISIB40 were significantly higher (*P* > 0.05) in the presence of glycerol than in its absence, while there were no differences in CFU·g^-1^ when different voltages were applied in both rhizobacteria tested (Table 1). The immobilization process only induced a significant reduction (*P* < 0.05) in the number of viable cells (~ 1.5 log units) of *P*. *agglomerans* ISIB55 and *B*. *caribensis* ISIB40 CFU·g^-1^ in the absence of glycerol. Therefore, the survival rates of *P*. *agglomerans* ISIB55 (0.98 ± 0.006 with 12 kV and 0.97 ± 0.01 with 22 kV) and *B*. *caribensis* ISIB40 (0.97 ± 0.05 with 12 kV and 0.92 ± 0.02 with 22 kV) in the presence of glycerol were significantly higher (*P* < 0.05) than those obtained in the same conditions in the absence of glycerol (*P*. *agglomerans* ISIB55, 0.83 ± 0.02 with 12 kV and 0.82 ± 0.01 with 22 kV; and *B*. *caribensis* ISIB40, 0.80 ± 0.01 with 12 kV and 0.78 ± 0.01 with 22 kV) (Table 1).

**Table 1:** Viability of *P*. *agglomerans* ISIB55 and *B*. *caribensis* ISIB40 during the immobilization process in nanofibers produced by the electrospinning technique.

Strain	Culture condition^a^	Electrospinning condition^b^	log_10_ CFU·g spinnig solution^-1^ pre-immobilization^c^	log_10_ CFU·g nanofiber^-1^ post-immobilization^d^	Survival rate (NAI·NBI^-1^)^e^
***P*. *agglomerans* ISIB55**	Pure	12 kV	8.99 ± 0.19	7.42 ± 0.02^h*^	0.83 ± 0.02^h^
		22 kV		7.39 ± 0.28^h*^	0.82 ± 0.01^h^
	with 5% glycerol	12 kV	9.08 ± 0.07	8.91 ± 0.10^g^	0.98 ± 0.006^g^
		22 kV		8.86 ± 0.15^g^	0.97 ± 0.01^g^
***B*. *caribensis* ISIB40**	Pure	12 kV	9.23 ± 0.13	7.45 ± 0.18^h*^	0.80 ± 0.01^h^
		22 kV		7.23 ± 0.15^h*^	0.78 ± 0.01^h^
	with 5% glycerol	12 kV	9.20 ± 0.15	8.99 ± 0.16^g^	0.97 ± 0.005^g^
		22 kV		8.74 ± 0.08^g^	0.95 ± 0.02^g^

Survival of rhizobacteria subjected to different electrospinning conditions.

^a^Bacterial culture conditions (with or without glycerol) employed to carry out the immobilization process.

^b^Voltage applied (12 kV or 22 kV) during the electrospinning technique.

^c^The data represent the average values of viable cells (log_10_ CFU·g^-1^ ± standard error) of *P*. *agglomerans* ISIB55 and *B*. *caribensis* ISIB40 from spinning solutions employed in three independent experiments before the immobilization process.

^d^The data represent the average values of viable cells (log_10_ CFU·g^-1^ ± standard error) of *P*. *agglomerans* ISIB55 and *B*. *caribensis* ISIB40 from nanofibers obtained in three independent experiments after the immobilization process. Statistically significant differences between viable cells pre and post-immobilization are indicated by * (*P* < 0.05). Different letters (g and h) indicate statistically significant differences (*P* < 0.05) in the log_10_ CFU·g^-1^ nanofiber between the different conditions evaluated during immobilization for each bacterial strain.

^e^Survival rate during immobilization expressed as NAI·NBI^-1^, where NAI and NBI are the log_10_ CFU·g^-1^ after and before immobilization, respectively. Different letters (g and h) indicate statistically significant differences (*P* < 0.05) in the survival rate between the different conditions evaluated during immobilization for each bacterial strain.

### Characterization of nanofibers containing beneficial rhizobacteria

On the basis of the viability results from the different immobilization conditions assayed, the nanofibers obtained from the bacterial culture-polymer mix subjected to electrospinning in the presence of 5% glycerol and 22 kV voltage level were selected for characterization. The electrospun voltage level of 22 kV was chosen because the amount of nanofibers obtained was higher than that obtained at 12 kV (data not shown), which facilitated nanofiber collection.

#### Morphology and size of nanofibers

The electrospun nanofibers with immobilized *P*. *agglomerans* ISIB55 and *B*. *caribensis* ISIB40 had a diameter of 543.18 ± 21.79 nm and 619.23 ± 36.89 nm, respectively, with no bead formation along them (S2A and S2B Fig).

#### Production of IAA, phosphate solubilization, siderophore synthesis and nitrogen fixation

**Fig 1:**
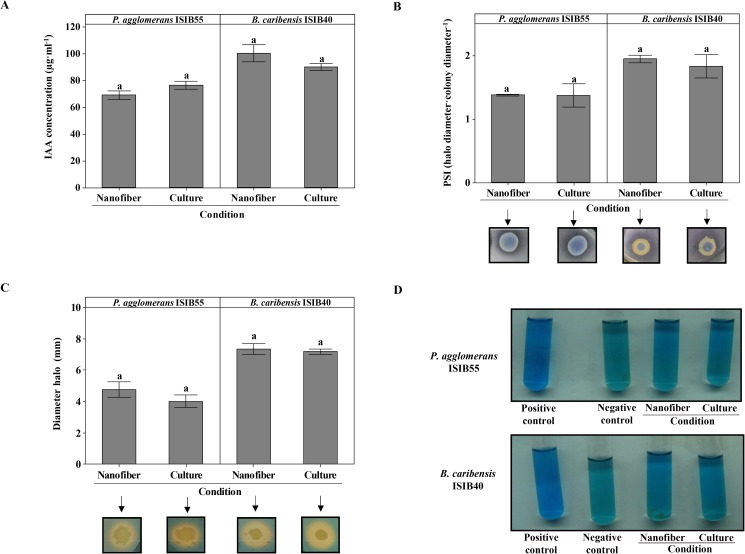
Beneficial properties of *P*. *agglomerans* ISIB55 (ISIB55) and *B*. *caribensis* ISIB40 (ISIB40). (A) Indole-3-acetic acid (IAA) production from nanofiber-immobilized cultures (nanofiber) and fresh cultures (culture) of ISIB55 and ISIB40. The data express the mean values ± standard error of IAA concentration expressed as μg·ml^-1^. (B) Phosphate solubilization from nanofiber and culture of ISIB55 and ISIB40. The data express the mean values ± standard error of phosphate solubilization index (PSI). The images below the bars show clear halos around the ISIB55 and ISIB40 colonies, indicating solubilizing capacity of phosphate in the different conditions evaluated. (C) Siderosphore synthesis from nanofiber and culture of ISIB55 and ISIB40. The data express the mean values ± standard error of halo diameters. The images below the bars show the color change from blue to orange that appeared around the colonies producing siderophores in the CAS-blue agar medium. Different letters in the graphs indicate statistically significant differences (*P* < 0.05) in IAA production, phosphate solubilization or siderosphore synthesis between the different conditions assayed for each bacterial strain, in accordance with Tukey's test. (D) Nitrogen fixation from nanofiber and culture of ISIB55 and ISIB40. The imagen shows the color change in the NFb semisolid medium when the bacteria fixed the nitrogen. *A*. *brasilense* Az39 and *E*. *coli* BW25113 were used as positive and negative controls, respectively.

In order to determine if the bacteria immobilized within the nanofibers maintained the beneficial properties associated with plant growth promotion, the abilities of *P*. *agglomerans* ISIB55 and *B*. *caribensis* ISIB40 to produce IAA, solubilize phosphate, synthesize siderophore and fix nitrogen were determined. Fig 1 shows that the beneficial properties of both rhizobacteria were maintained when their cultures were embedded in electrospun nanofibers. No significant differences (*P* > 0.05) were observed in the different biochemical markers associated with plant growth promotion between nanofiber-immobilized cells and planktonic cells in culture (Fig 1).

### Characterization of seeds coated with nanofiber-immobilized rhizobacteria

#### Rhizobacteria survival in soybean seeds

**Fig 2:**
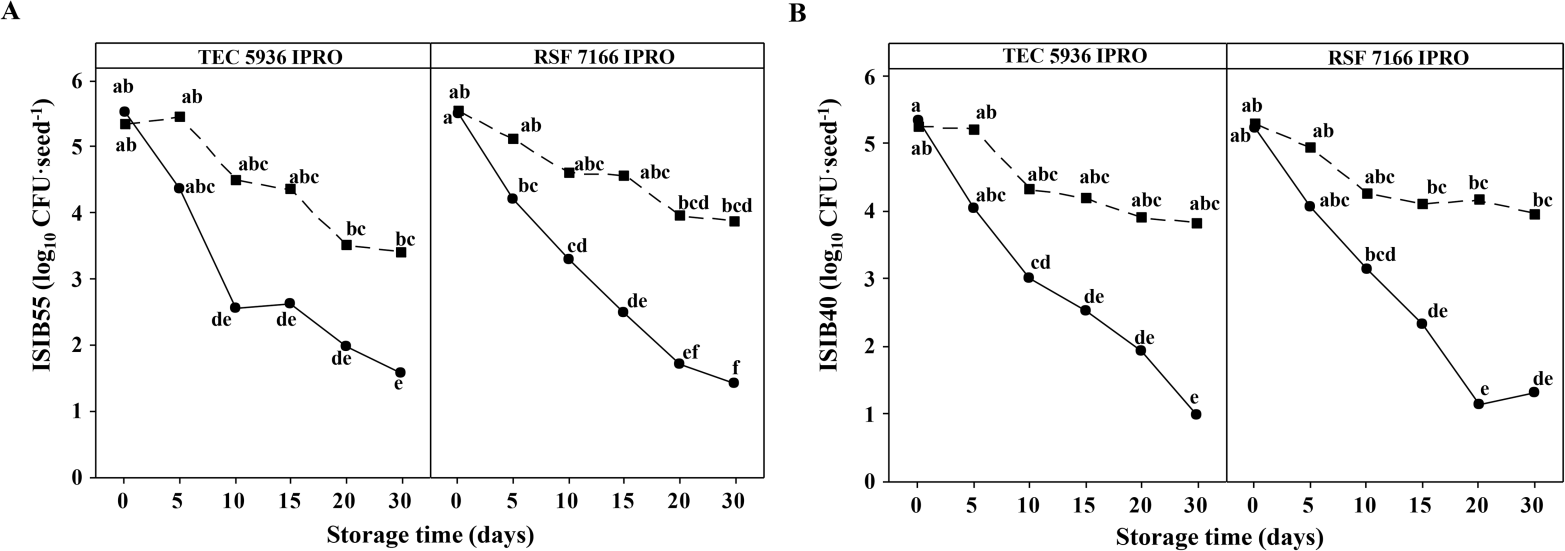
Viability of *P*. *agglomerans* ISIB 55 (ISIB55) and *B*. *caribensis* ISIB40 (ISIB40) in soybean seeds (TEC 5936 IPRO and RSF 7166 IPRO varieties). (A) Seeds treated with (■) nanofiber-immobilized ISBI55 or (●) ISBI55 culture; and (B) seeds treated with (■) nanofiber-immobilized ISBI40 or (●) ISBI40 culture. After treatment, the seeds were stored for 30 days at room temperature. Data are plotted as the mean values of viable cell numbers (log_10_ CFU·seed^-1^). Different letters indicate statistically significant differences (*P* < 0.05) in the number of viable cells between seed treatment, seed variety and storage time, in accordance with Tukey's test.

The survival of *P*. *agglomerans* ISIB55 and *B*. *caribensis* ISIB40 was studied in ISIB55-PVA or ISIB40-PVA-treated seeds and ISIB55 or ISIB40-treated seeds. Fig 2 shows that bacterial viability was higher in the seeds coated with nanofiber-immobilized rhizobacteria than in the seeds inoculated with the bacterial cultures. A general decrease in the viability of *P*. *agglomerans* ISIB55 and *B*. *caribensis* ISIB40 was observed throughout the storage time (Fig 2). However, this survival decrease was only significant (*P* < 0.05) in the seeds inoculated with bacterial cultures. For the ISIB55-treated seeds, a significant reduction in viability (~ 2.5 log_10_ CFU·seed^-1^) was evidenced from day 10 of storage in the two seed varieties evaluated (TEC 5936 IPRO and RSF 7166 IPRO) (Fig 2A). Similarly, in the ISIB40-treated seeds, a significant diminution was observed from days 10 and 15 in the seeds of the TEC 5936 IPRO and RSF 7166 IPRO varieties, respectively (Fig 2B).

#### Soybean seed appearance

**Fig 3:**
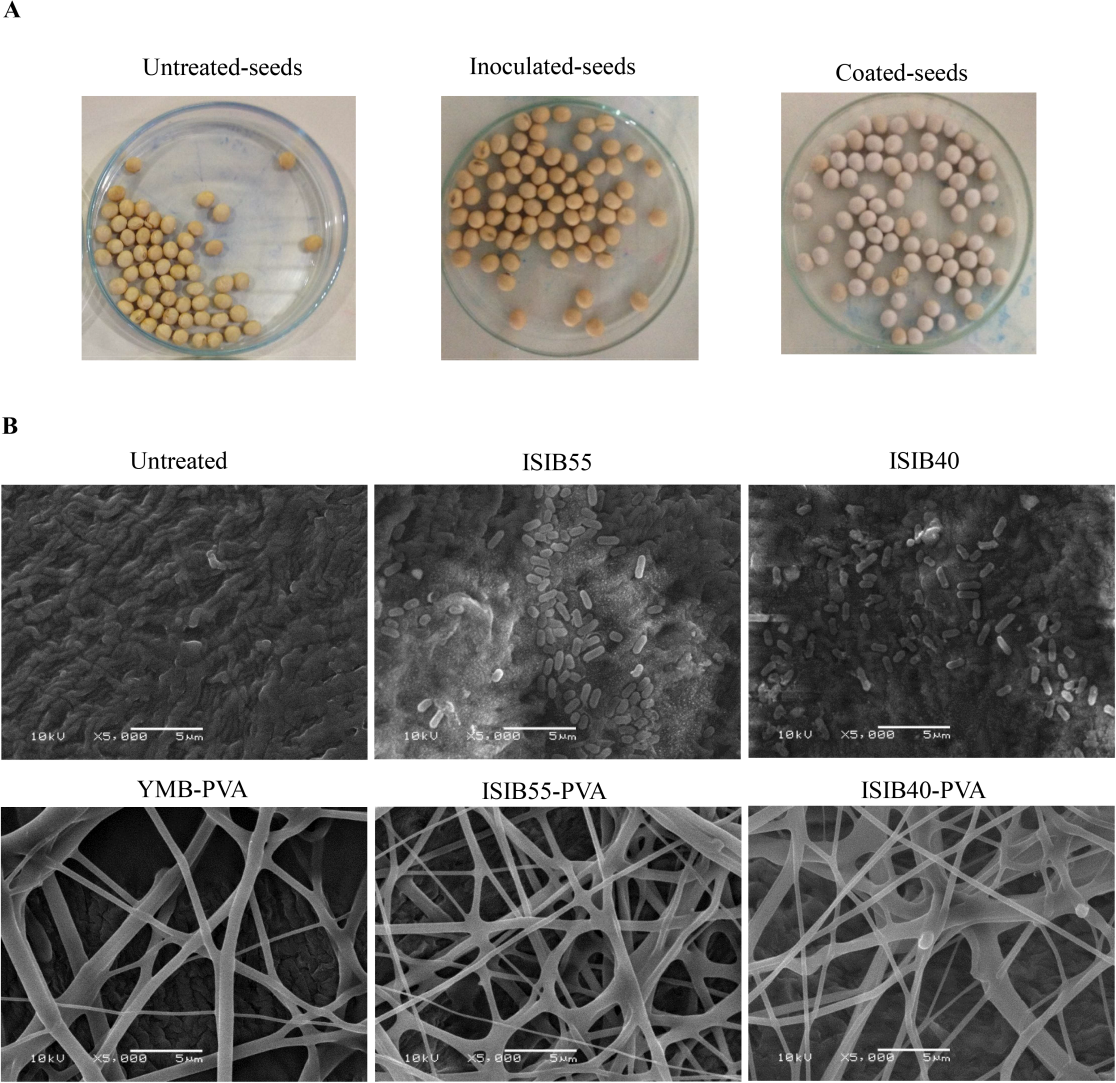
Macro and micro-appearance of soybean seeds (TEC 5936 IPRO variety). (A) Photographs of soybean seeds not treated (Untreated-seeds), inoculated with rhizobacteria cultures (Inoculated-seeds), and coated with nanofiber-immobilized rhizobacteria (Coated-seeds). (B) Scanning electron microscopy micrographs of untreated seeds (Untreated), inoculated with *P*. *agglomerans* ISIB55 (ISIB55) or *B*. *caribensis* ISIB40 (ISIB40) cultures and coated with nanofiber-immobilized Yeast Mannitol Broth medium (YMB-PVA), nanofiber-immobilized ISIB55 (ISIB55-PVA) or nanofiber-immobilized ISIB40 (ISIB40-PVA). The micrographs were observed at 5000x magnification. The results are representative of two independent experiments. Similar results were observed in the seed variety RSF 7166 IPRO.

Both seed coating with nanofiber-immobilized rhizobacteria and seed inoculation with bacterial culture resulted in seeds with good appearance. The seeds coated with the bacterial culture-polymer mix evidenced a film homogeneously adhered onto the seed surface, while those inoculated with rhizobacterial cultures did not present macroscopic differences compared with the untreated seeds (Fig 3A). The presence of nanofibers and rhizobacteria on the seed surface was verified by SEM micrographs (Fig 3B). Fig 3B shows the surface of the untreated seeds, ISIB55 or ISIB40-treated seeds, and nanofibers deposited on the surface of the YMB-PVA, ISIB55-PVA or ISIB40-PVA-treated seeds. The presence of embedded rhizobacteria in the electrospun PVA nanofibers was not evidenced by SEM, which could be due to the fact that the nanofiber diameters were higher than the bacterial diameters (Fig 3B).

#### Soybean germination

When the soybean seeds (TEC 5936 IPRO and RSF 7166 IPRO varieties) were subjected to the germination test, no significant differences were observed in any of the parameters evaluated (germination index and rate, root and shoot length, vigor index) between the YMB, YMB-PVA, ISIB55, ISIB40 and ISIB40-PVA seeds. This demonstrates that the seed coating process with PVA or *B*. *caribensis* ISIB40-PVA mix and seed inoculation with *P*. *agglomerans* ISIB55 and *B*. *caribensis* ISIB40 cultures did not affect soybean seed germination (Table 2). However, a significant increase (*P* < 0.05) in the germination rate was observed in the seeds (TEC 5936 IPRO variety) coated with nanofiber-immobilized *P*. *agglomerans* ISIB55 (98.9 ± 1.1) compared with both the YMB and YMB-PVA seeds (91.8 ± 2.4 and 92.9 ± 2.0, respectively). Similarly, in the RSF 7166 IPRO variety seeds, maximal germination rate was observed in the ISIB55-PVA-treated seeds (86.9 ± 3.4) compared to all the seed treatments, but no significant differences (*P* > 0.05) were evidenced (Table 2).

**Table 2:** Effects of seed coating with nanofiber-immobilized beneficial rhizobacteria on germination, seedling growth and vigor index of soybean.

	Seed variety	Seed treatment					
		YMB	YMB-PVA	ISIB55	ISIB55-PVA	ISIB40	ISIB40-PVA
**Germination index**^c^	TEC 5936 IPRO	18.3 ± 0.9^a^	19.9 ± 0.5^a^	19.4 ± 1.7^a^	18.6 ± 1.1^a^	19.6 ± 1.3^a^	19.5 ± 1.3^a^
	RSF 7166 IPRO	14.6 ± 1.3^a^	15.6 ± 0.9^a^	14.6 ± 1.7^a^	16.1 ± 1.3^a^	14.7 ± 1.1^a^	16.2 ± 1.3^a^
**Germination rate (%)**^d^	TEC 5936 IPRO	91.8 ± 2.4^b^	92.9 ± 2.0^b^	93.9 ± 2.4^ab^	98.9 ± 1.1^a^	94.9 ± 2.1^ab^	95.9 ± 2.6^ab^
	RSF 7166 IPRO	75.5 ± 3.4^a^	80.6 ± 5.3^a^	79.6 ± 5.3^a^	86.9 ± 3.4^a^	76.5 ± 5.1^a^	81.6 ± 2.6^a^
**Root + shoot length (cm)**	TEC 5936 IPRO	4 ± 0.1^ab^	3.9 ± 0.3^ab^	3.7 ± 0.2^b^	4.5 ± 0.3^a^	4.4 ± 0.3^ab^	4 ± 0.3^ab^
	RSF 7166 IPRO	2.8 ± 0.2^ab^	2.8 ± 0.3^ab^	2.7 ± 0.2^b^	3.4 ± 0.3^a^	2.8 ± 0.2^ab^	3.2 ± 0.2^ab^
**Vigor index**^e^	TEC 5936 IPRO	371.8 ± 17.9^b^	353.1 ± 10.4^b^	374.1 ± 38.8^ab^	449.9 ± 31.6^a^	422.5 ± 33.9^ab^	382.4 ± 33.3^ab^
	RSF 7166 IPRO	214.3 ± 18.6^b^	236.5 ± 9.2^ab^	219.1 ± 29.6^ab^	302.6 ± 32^a^	226.9 ± 26.4^ab^	263.6 ± 25.5^ab^

Values are the mean of two independent experiments with four replicates obtained from seeds: inoculated with Yeast Manitol Broth (YMB), coated with nanofiber-immobilized YMB (YMB-PVA), nanofiber-immobilized *P*. *agglomerans* ISIB55 or *B*. *caribensis* ISIB40 cultures (ISIB55-PVA and ISIB40-PVA, respectively), and inoculated with *P*. *agglomerans* ISIB55 (ISIB55) or *B*. *caribensis* ISIB40 (ISIB40) cultures. Mean values ± standard error within a row followed by different letters (a and b) are significantly different (*P* < 0.05).

^c^*Germination index* = ∑ *number of germinated seeds on day N/N days of seed germination*.

^d^ *Germination rate* (*GR*) = (*number of germinated seeds/total seed number used in the test*) × 100.

^e^*Vigor index* = (*mean root length* + *mean shoot length*) × *GR*.

On the other hand, in both seed varieties tested, coating with ISIB55-PVA did not affect the total length of roots plus shoots compared with the YMB and YMB-PVA seeds. Nevertheless, coating with ISIB55-PVA significantly increased (*P* < 0.05) the total length of root plus shoot compared with the ISIB55-treated seeds (Table 2).

The increase in the germination rates in both seed varieties coated with ISIB55-PVA conducted to a significant augment (*P* < 0.05) in the vigor index (449.9 ± 31.6 and 302.6 ± 32 in the TEC 5936 IPRO and RSF 71 66 IPRO varieties, respectively) compared to the YMB-treated seeds (371.8 ± 17.9 and 214.3 ± 18.6 in TEC 5936 IPRO and RSF 7166 IPRO, respectively) and the YMB-PVA-treated seeds (only in TEC 5936 IPRO variety, 353.1 ± 10.4) (Table 2). These results indicate that only the immobilization of *P*. *agglomerans* ISIB55 in the nanofibers improved the beneficial properties of the isolate, as evidenced by the significant increase in the germination rate and vigor index in the soybean seeds.

### Colonization of soybean roots by rhizobacteria

**Fig 4:**
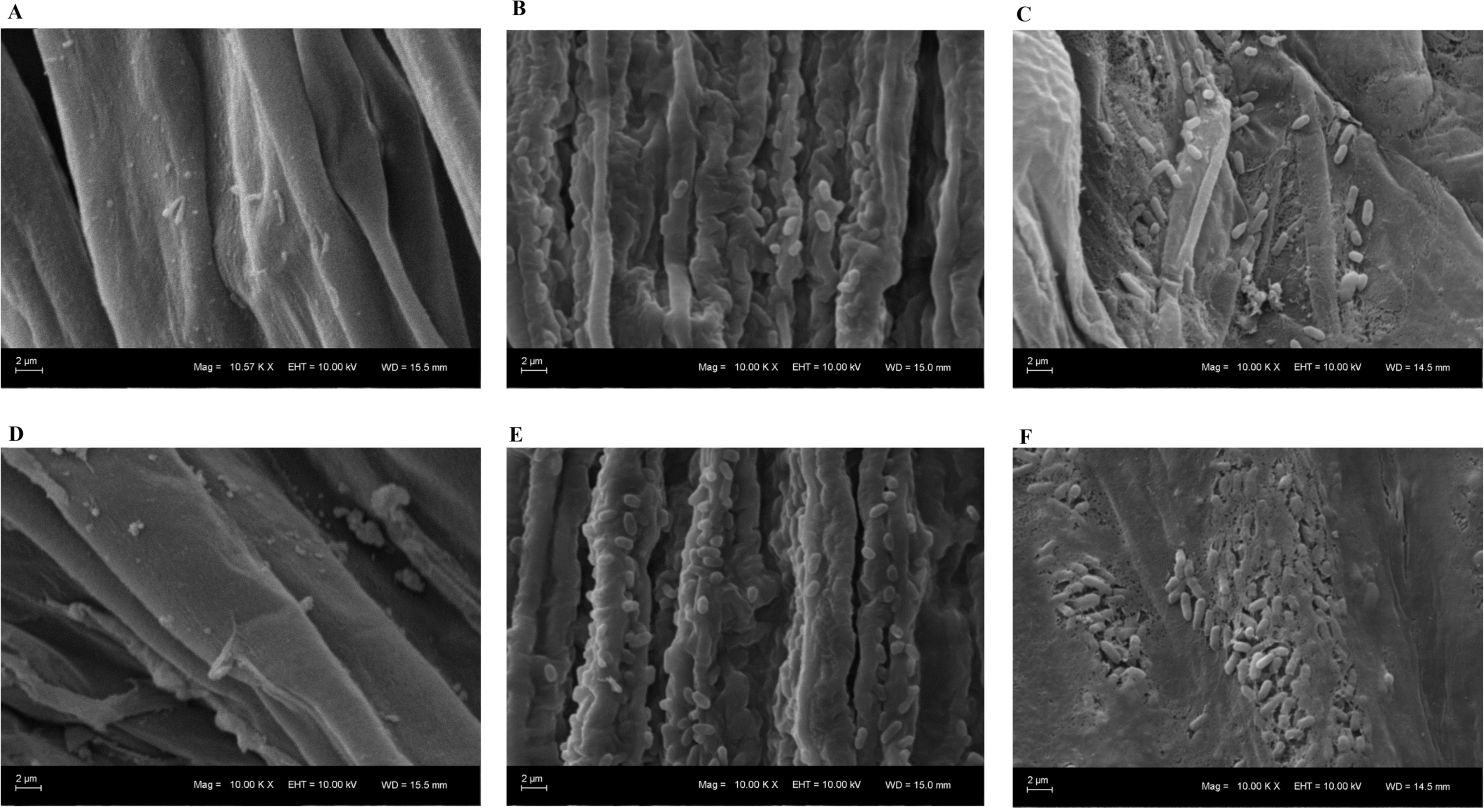
Scanning electron microscopy micrographs of soybean roots from seeds (TEC 5936 IPRO variety). Seeds (A) untreated, (B) inoculated with *P*. *agglomerans* ISIB55 (ISIB55) or (C) *B*. *caribensis* ISIB40 (ISIB40) cultures and (D) coated with nanofiber-immobilized Yeast Mannitol Broth medium (YMB-PVA), (E) nanofiber-immobilized ISIB55 (ISIB55-PVA) or (F) nanofiber-immobilized ISIB40 (ISIB40-PVA). Images observed at 5000× magnification show a visibly greater colonization of rhizobacteria on the roots from the coated seeds compared to the inoculated seeds. Similar results were observed in the seed variety RSF 7166 IPRO. The results are representative of two independent experiments.

The ability of *P*. *agglomerans* ISIB55 and *B*. *caribensis* ISIB40 to colonize soybean roots from treated seeds was evaluated. Scanning electron microscopy revealed that soybean roots from seeds subjected to a) coating with ISIB55-PVA or ISIB40-PVA and b) inoculation with ISIB55 or ISIB40 culture were successfully colonized by both rhizobacteria (Fig 4B, 4C, 4E and 4F). Fig 4 shows a visibly higher microorganism colonization on the soybean roots with seeds coated with nanofiber-immobilized rhizobacteria compared to seeds inoculated with bacterial cultures. As expected, bacterial presence was not evidenced in the roots from the untreated and YMB-PVA seeds (Fig 4A and 4D).

#### Growth promotion in soybean

**Fig 5:**
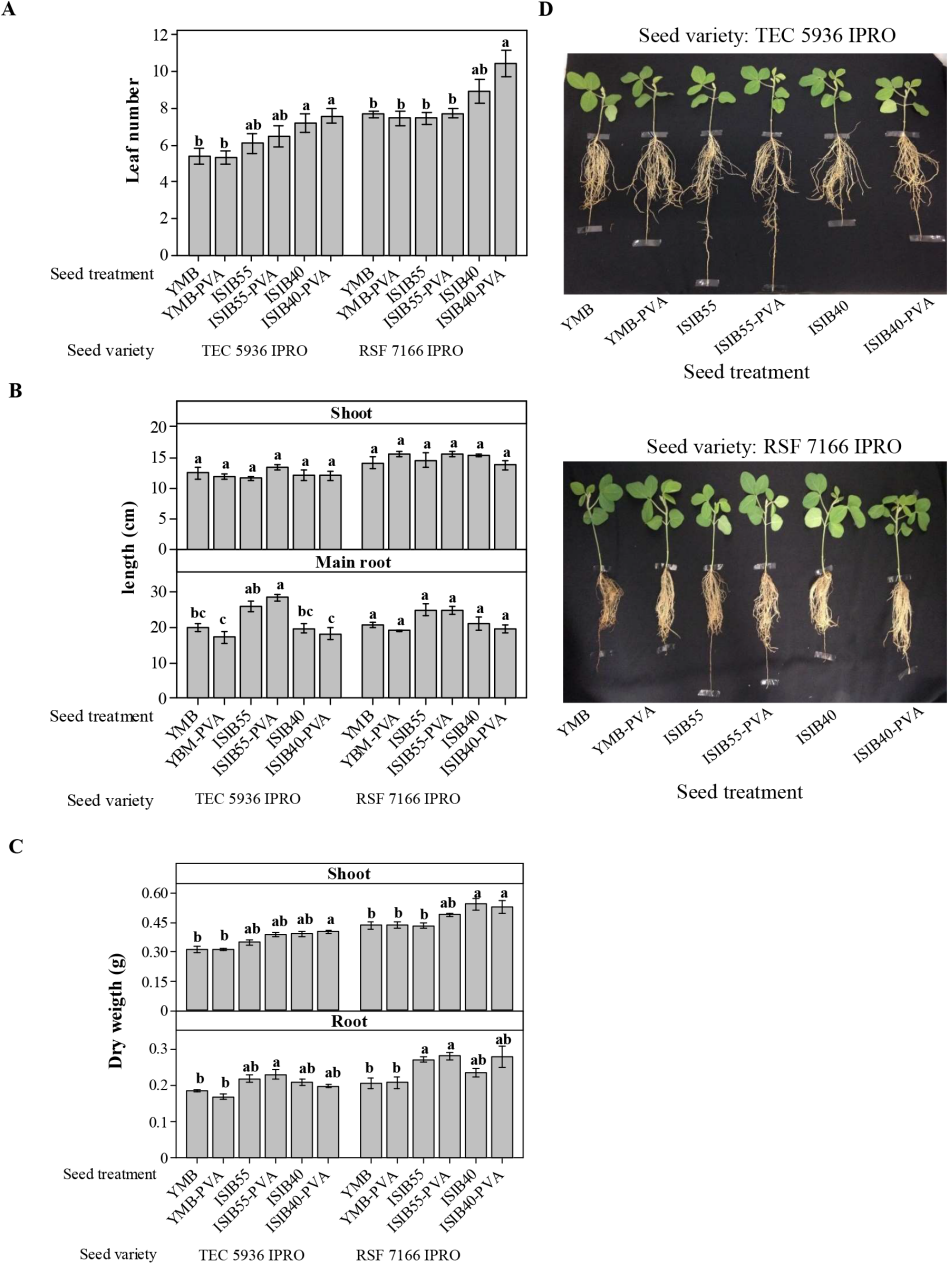
Effect of seed coating with nanofiber-immobilized rhizobacteria on growth promotion in soybean plants. (A) Leaf number, (B) length of shoot and main root and (C) dry weight of shoot and root determined in plants from seeds (TEC 5936 IPRO and RSF 7166 IPRO varieties) inoculated with Yeast Mannitol Broth medium (YMB), coated with nanofiber-immobilized YMB (YMB-PVA), nanofiber-immobilized *P*. *agglomerans* ISIB55 (ISIB55-PVA) or nanofiber-immobilized *B*. *caribensis* ISIB40 (ISIB40-PVA) and inoculated with *P*. *agglomerans* ISIB55 (ISIB55) or *B*. *caribensis* ISIB40 (ISIB40) cultures. Data are plotted as mean values ± standard error. Different letters indicate statistically significant differences between seed treatments in the same variety (*P* < 0.05). (D) Photographs of soybean plants (TEC 5936 IPRO and RSF 7166 IPRO varieties) obtained after 25 days of seed seeding treated, as described above. Results are representative of two independent experiments.

The evaluation of the indicator parameters of plant growth promotion demonstrates that seed coating with ISIB40-PVA significantly increased leaf number compared to the YMB and YMB-PVA-treated seeds in both seed varieties assayed. The plants from the ISIB40-treated seeds also showed a higher leaf number than those from the YMB and YMB-PVA seeds, but the beneficial effect was more evident in the plants from the ISIB40-PVA-treated seeds (Fig 5A and 5D). Moreover, in both seed varieties, coating and inoculation with *P*. *agglomerans* ISIB55 did not modify leaf number in comparison with the controls.

On the other hand, shoot length was not affected by any of the treatments, whereas an increase in main root length was induced with the inoculation and coating of the seeds with *P*. *agglomerans* ISIB55 (ISIB55 and ISIB55-PVA-treated seeds, respectively) (Fig 5B and 5D). Significant differences between the treatments were evidenced only in the TEC 5936 IPRO variety. The main root length of the plants from the ISIB55-PVA-treated seeds was significantly higher compared to the plants from all the treatments, with the exception of those from the ISIB55-treated seeds (Fig 5B).

When analyzing the dry matter in both soybean varieties, the plants from the ISIB40-PVA and ISIB50-PVA seeds evidenced a significantly higher weight of shoot and root (*P* < 0.05) respectively, compared to the plants from the YMB and YMB-PVA seeds (Fig 5C). Moreover, similar results were only evidenced in the plants from the RSF 7166 IPRO variety seeds inoculated with both *B*. *caribensis* ISIB40 and *P*. *agglomerans* ISIB55 (Fig 5C).

The obtained results in this stage indicate that the beneficial effect of *P*. *agglomerans* ISIB55 and *B*. *caribensis* ISIB40 on the different growth parameters was increased with the bacterial immobilization process in the nanofibers.

## Discussion

The inoculation of legume seeds with nitrogen-fixing symbiont bacteria, such as *Rhizobium* and *Bradyrhizobium*, is a common practice that has been implemented in the agriculture of several countries [39–42]. Rhizobia inoculation maximizes legume yield due to the ability of these microorganisms for nodular and nitrogen fixing, which is highly required for grain yield [15]. However, legume nutrient acquisition can also be promoted by other microorganisms that contribute to phosphorus and nitrogen uptake [4,43]. Linu *et al*. [43] reported that inoculation of cowpea with phosphate solubilizing *Burkholderia* spp. improved nodulation, root and shoot biomass, straw and grain yield and phosphate and nitrogen uptake by the crop. Moreover, several studies have clearly demonstrated that co-inoculation of PGPR microorganisms and *Bradyrhizobium* significantly improved soybean growth and its yield with respect to the application of *Bradyrhizobium* by itself. Therefore, considering that other PGPR genera besides *Bradyrhizobium*, can also exert beneficial effects on soybean growth, their evaluation concerning the improvement of plant performance is also of great importance. In the present work, two potential PGPR strains, *P*. *agglomerans* ISIB55 and *B*. *caribensis* ISIB40, were employed to evaluate the impact of soybean seed coating with nanofiber-immobilized PGPR on different plant growth parameters. Similarly to the present study, both bacterial genera have been described in the literature as potential PGPR through their abilities to solubilize phosphate, produce IAA, synthetize siderosphore and/or fix nitrogen [43–45].

Electrospinning, a very useful and effective technique for the manufacture of polymeric nanofibers is used for a variety of biomedical and biotechnological applications, such as drug delivery, catalysis, tissue engineering and chemical and biological sensors [46–49]. In addition, electrospinning has recently been employed to coat soybean seeds with nanofiber-immobilized *Bradyrhizobium* strains. This process improved bacterial survival in seeds stored at room temperature for 48 h and increased the number of nodules formed in soybean plants [27]. Currently, the work of Damasceno *et al*. [27] is the only report in the literature that employs electrospinning to immobilize beneficial microorganism on soybean seeds. Despite promising results, further studies are required to evaluate other PGPR genera that could be immobilized using this methodology. Electrospinning conditions that improve microorganism survival and seed characterization after the process need to be assessed.

In the present work, different conditions of electrospinning were tested for immobilized *P*. *agglomerans* ISIB55 and *B*. *caribensis* ISIB40. The condition that allowed a better survival of the rhizobacteria was used to the soybean seed coating with nanofiber-immobilized rhizobacteria. Following this, the characterization of the seeds coated with nanofiber-immobilized rhizobacteria was carried out through the evaluation of bacterial survival during seed storage, germination and plant growth.

PVA polymer was used to immobilize *P*. *aggomerans* ISIB55 and *B*. *caribensis* ISIB40 on the basis of the results obtained in other studies in which different bacterial strains were successfully immobilized with the aforementioned polymer [27,37,38]. PVA is a biodegradable hydrophilic polymer, generally recognized as safe (GRAS), which has a high oxygen barrier when dry without disturbing bacterial bioactivity [50]. The exposure of *P*. *aggomerans* ISIB55 and *B*. *caribensis* ISIB40 to PVA had no effect on their viability, even when rhizobacteria remained in this solution for several days before testing (data not shown). Immediately after electrospinning with the bacterial culture-PVA mix, high viability percentages were recovered in both bacteria (*P*. *aggomerans* ISIB55, ~ 82%; *B*. *caribensis* ISIB40 ~ 80%, Table 1) in the two voltage conditions (12 and 22 kV) assayed. In addition, under the above conditions, higher viability percentages (*P*. *aggomerans* ISIB55, ~ 98%; *B*. *caribensis* ISIB40 ~ 95%, Table 1) were evidenced with the addition of 5% glycerol, indicating that immobilization of microorganisms was efficient and that glycerol conferred a protective effect against immobilization-induced stress. In agreement with the above, Salalha *et al*. [37] reported that glycerol caused a substantial increase in the viability of *Escherichia coli* when the cells were subjected to electrospinning. These authors suggest that glycerol can enter bacteria and protect them from the fast dehydration that occurs when nanofibers are produced. On the other hand, a voltage of 22 kV during the process was selected in the present study, considering that no difference in rhizobacterial survival was found between the two voltages evaluated.

During the electrospinning process, the characteristics of nanofibers can be modified by numerous variables, such as solution properties and process parameters. Electrospinning studies employing PVA have demonstrated that higher voltages yield larger fiber diameters [51]. In this work, a greater quantity of nanofibers was recovered with 22 kV compared to 12 kV (data not shown). Furthermore, 22 kV application during electrospinning resulted in nanofiber diameters of 543.18 ± 21.79 and 619.23 ± 36.89 nm in the immobilization of *P*. *agglomerans* ISIB55 and *B*. *caribensis* ISIB40, respectively (S2A and S2B Fig). By applying lower voltages, Salalha *et al*. [37] and Fung *et al*. [38] obtained smaller nanofiber diameters (ranging between 229 and 400 nm) to encapsulate *E*. *coli* and *Lactobacillus acidophilus*, respectively. Additionally, these authors, using SEM, found bacterial cells embedded in electrospun PVA nanofibers. In contrast, in this study it was not possible to find them, probably due to the larger nanofiber diameters obtained. Nevertheless, microbiological studies demonstrated the presence of viable cells of *P*. *agglomerans* ISIB55 and *B*. *caribensis* ISIB40 in the nanofibers (Table 1).

Because *P*. *agglomerans* ISIB55 and *B*. *caribensis* ISIB40 remained viable after immobilization, the expression of the potential beneficial characteristics of both immobilized rhizobacteria was evaluated. The ability to produce IAA, solubilize phosphate, synthetize siderosphore and fix nitrogen of *P*. *agglomerans* ISIB55 and *B*. *caribensis* ISIB40 was maintained when these microorganisms were immobilized (Fig 1). This demonstrates that electrospinning preserved the rhizobacteria in viable and physiologically intact conditions.

There are studies that clearly highlight the relationship between the number of bacteria applied on seeds and crop yields [52,53]. Therefore, quality standards of inoculants, which vary slightly between countries, have established that the amount of microorganisms required ranges from 5 x 10^7^ to 1 x 10^9^ CFU·g^-1^ or ml^-1^ inoculant. Another approach to the question of inoculant standards considers the minimum number of viable cells per seed after application at the manufacturers’ recommended rate. The minimum quantities of rhizobacteria accepted per seed are 10^3^ for small, 10^4^ for medium and 10^5^ for large seeds, such as soybean [54,55]. In the present work, soybean seed coating was carried out with a bacterial culture-polymer mix containing approximately 10^9^ CFU·g spinning solution^-1^ (Table 1). Immediately after seed coating by electrospinning, the number of viable cells per seed obtained was approximately 10^5^ CFU for both *P*. *agglomerans* ISIB55 and *B*. *caribensis* ISIB40 in the two seed varieties tested (Fig 2).

A major problem in the inoculant industry is the reduction in microorganism viability when inoculants come in contact with seeds and during seed storage. Inhibitory exudates present in the seed tegument, desiccation of support, natural seed microflora and environmental stress (*e*.*g*. oxygen content, temperature and humidity) are some of the factors that may affect bioinoculant viability [4,16,55]. It was observed that the seeds (TEC 5936 IPRO and RSF 7166 IPRO varieties) inoculated with cultures of *P*. *agglomerans* ISIB55 or *B*. *caribensis* ISIB40 evidenced a significant decrease in the viability of both rhizobacteria during the 30 days of storage at room temperature. However, the reduction in viability was minimized when the seeds were coated with nanofiber-immobilized rhizobacteria (Fig 2A and 2B). A possible reason for the above is that the polymer was capable of creating a microenvironment around the cells, thus limiting access to toxic substances and controlling environmental factors.

It has been shown in this study that only the seed coating with ISIB55-PVA improved the germination rate (in the TEC 5936 IPRO variety) and vigor (in both seed varieties studied) compared with the control seeds (YMB and YMB-PVA). Improvement in seed germination parameters by rhizobacteria has been reported in several crops [36,56,57]. However, inhibition of *in vitro* soybean germination by PGPR, probably due to nutrient competition between seeds and bacteria, has also been reported [34,58]. In this work, seed inoculation with rhizobacteria cultures did not show changes in any of the evaluated parameters in an *in vitro* soybean germination assay. Thus, we suggest that the improvement in germination in ISIB55-PVA seeds could be due to the fact that PVA acts as a substrate for *P*. *agglomerans* ISIB55 growth. Because PVA is a biodegradable polymer that can be degraded by soil microorganism [59], it could act as a nutrient source.

From the results obtained with the soybean growth promotion assay in the two seed varieties assayed, no significant difference was evidenced between the seeds coated with nanofiber-immobilized rhizobacteria and those inoculated with bacterial culture. However, when comparing growth parameters with the YMB-PVA and YMB control seeds, better results were found with the seed coating with nanofiber-immobilized rhizobacteria. In general, in the two seed varieties, plants from the seeds coated with *B*. *caribensis* ISIB40 evidenced a significantly higher leaf number and dry weight of shoots while the plants from the seeds coated with *P*. *agglomerans* ISIB55 presented a significantly higher length and dry weight of root (Fig 5). These results could be correlated with the visibly greater colonization of rhizobacteria evidenced by SEM on the roots from the coated seeds compared to the inoculated seeds (Fig 4). Given that the colonization of roots by bacteria is an important step in the interaction between beneficial bacteria and the host plant [60,61], we suggest that the seed coating with rhizobacteria contributed to bacterial colonization in the roots, which positively impacted plant growth.

Although the use of *Burkholderia* spp. strains such as PGPR is limited by the risk they can cause to human health; this is mainly attributable to the *B*. *cepacia* complex species [62,63]. Frickmann *et al*. [64] and Pan *et al*. [65] carried out phylogenetic studies using *rpsU* and 16S rRNA gene sequences, respectively, and demonstrated that *B*. *caribensis* specie strains are not closely related to the *B*. *cepacia* complex species. Moreover, Paungfoo-Lonhienne *et al*. [63] reported *B*. *caribensis* strains, such as plant-beneficial-environmental *Burkholderia*. Taking the above references into account, it was concluded that the use of the *B*. *caribesis* species, such as a PGPR, could be biosafe. On the other hand, several works have also reported the potential of *B*. *caribensis* strains as PGPR [43,45,66,67]. Chen *et al*. [66] identified numerous strains of *B*. *caribensis*, such as nitrogen-fixing legume symbionts. Similarly, Roy *et al*. [67] and Parra-Cota *et al*. [45] demonstrated that *B*. *caribensis* strains promote growth and increase yield in rice and grain amaranth, respectively, by improving plant nitrogen uptake. In the present work, *B*. *caribensis* ISIB40 was also able to fix nitrogen (Fig 1D). Thus, this ability of *B*. *caribensis* ISIB40 could be suggested as one of the possible mechanisms that induced higher leaf number and dry weight of shoots in the plants from the ISIB40-PVA and ISIB40-treated seeds. However, further studies will be necessary to confirm this hypothesis.

The production of longer roots and higher numbers of root hairs and secondary roots in plants, which are involved in water and nutrient uptake, are associated with the indolic phytohormone [68,69]. It has been demonstrated here that both *B*. *caribenisis* ISIB40 and *P*. *agglomerans* ISIB55 presented the ability to produce IAA (Fig 1A). However, longer roots and higher dry weight of roots was only evidenced in the soybean plants from the ISIB55-PVA and ISIB55-treated seeds (Fig 5B, 5C and 5D). It has been reported that native isolates are previously adapted to the local environment, which results in a competitive advantage over non-natives strains [70]. Thus, the present results suggest that *P*. *agglomerans* ISIB55 (a soybean isolate) could present a better adaptation to exert its beneficial effects compared to *B*. *caribensis* ISIB40 (a sugar isolate).

Finally, it is important to highlight the novelty of the present work because this is the first study to report the germination and growth of soybean seeds coated with nanofiber-immobilized potential PGPR and to show promising results. Despite the fact that Damasceno *et al*. [27] also employed the same technique to coat soybean seeds with nanofiber-immobilized *Bradyrizhobium* strains, these authors only reported soybean nodulation and bacterial survival in seeds stored for 48 h. However, the present work, in addition to the soybean germination and growth results, reports an improvement in bacterial survival in seeds coated with nanofiber-immobilized rhizobacteria stored for 30 days. Thus, the present results highlight the potential of the electrospinning technique to coat seeds with PGPR. Further studies could be carried out to evaluate the effect of soybean seed coating with co-cultures of *Bradyrhizobium* and *P*. *agglomerans* ISIB55 or *B*. *caribensis* ISIB40 by the described methodology.

## Conclusions

The electrospinning technique proved effective in the encapsulation of *P*. *agglomerans* ISIB55 and *B*. *caribensis* ISIB40 since it did not affect either the viability or the beneficial properties of these rhizobacteria. Seed coating with nanofiber-immobilized rhizobacteria improved *P*. *agglomerans* ISIB55 and *B*. *caribensis* ISIB40 survival on the seeds stored for 30 days and contributed to the successful colonization of both bacteria on the plant root. Moreover, seed coating with *P*. *agglomerans* ISIB55 increased germination, length and dry weight of the root. On the other hand, seed coating with *B*. *caribensis* ISIB40 augmented leaf number and dry weight of the shoot. Therefore, the technique applied in the present work to coat seeds with nanofiber-immobilized PGPR could be considered a promising eco-friendly approach to improve soybean production through the use of a microbial inoculant. However, yield studies should be carried out to confirm the PGPR activity of *P*. *agglomerans* ISIB55 and *B*. *caribensis* ISIB40.

With the innovative process developed in this work, PGPR could exert their beneficial effect on soybean while the polymer coating protects bacteria and seeds from the abiotic stress of the environment and promotes successful inoculant colonization. Further studies should be carried out to develop this methodology on an industrial scale.

## Supporting information

S1 Fig Diagram summarizing the process employed to coat soybean seeds with nanofiber-immobilized rhizobacteria. (TIF)

S2 Fig Scanning electron microscopy micrographs of electrospun nanofibers. Nanofibers obtained from (A) *P*. *agglomerans* ISIB55-polymer mix and (B) *B*. *caribensis* ISIB40-polymer mix. The micrographs were observed at 5000x magnification. Results are representative of three independent experiments.
(TIF)
