# Supplementary figures and images for: Beneficial rhizobacteria immobilized in nanofibers for potential application as soybean seed bioinoculants
Source: PLoS One. 2017 May 4;12(5):e0176930. doi: 10.1371/journal.pone.0176930 (PMC5417607; doi:10.1371/journal.pone.0176930)

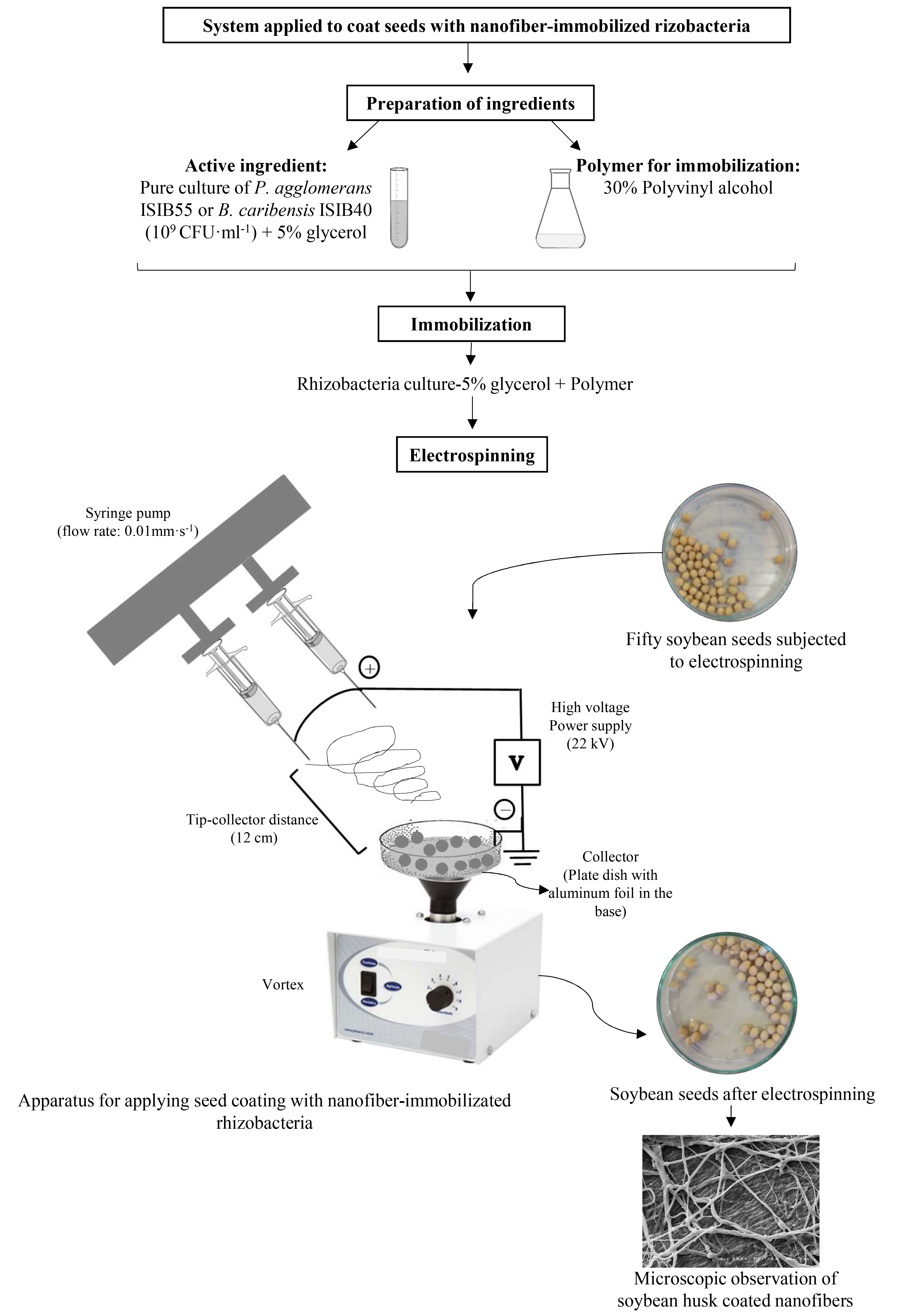

Supplement: S1 Fig — (TIF) [file pone.0176930.s001.tif]

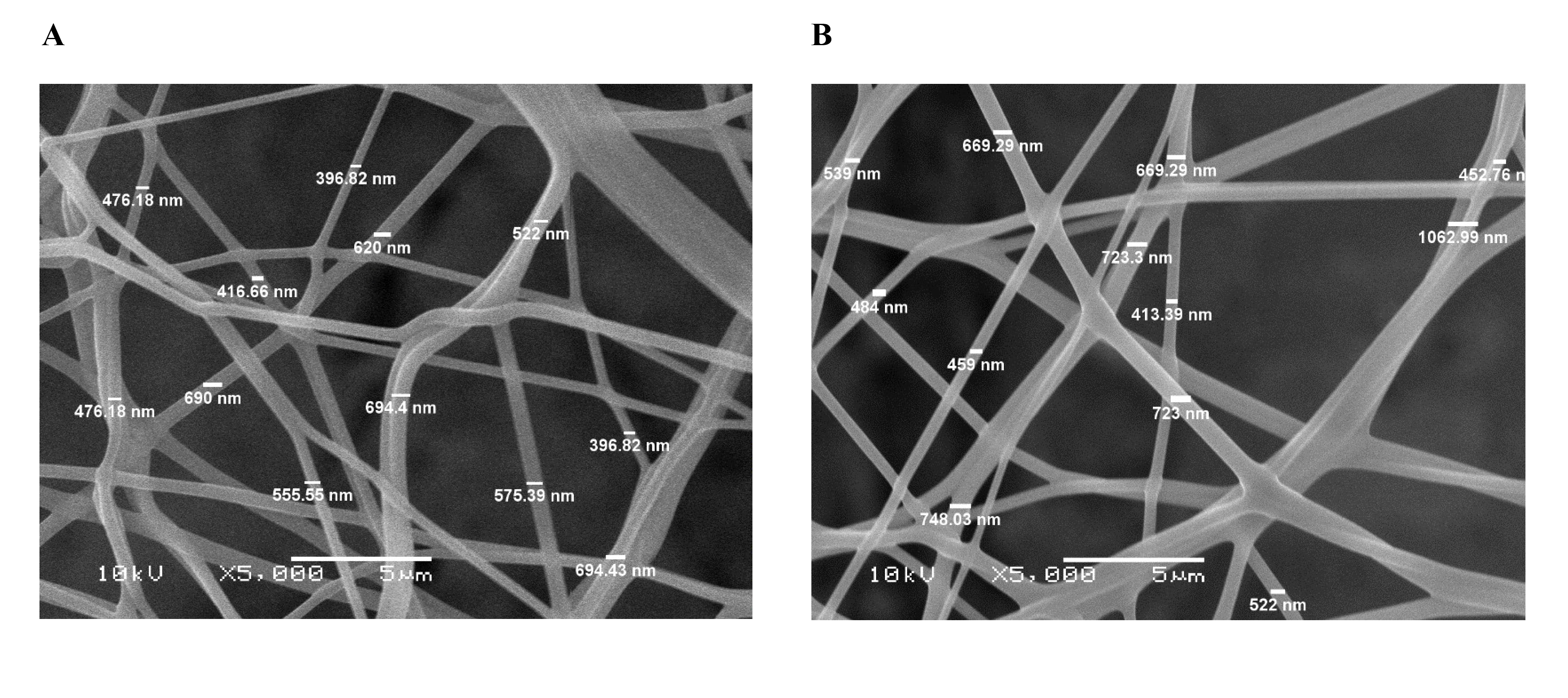

Supplement: S2 Fig — Nanofibers obtained from (A) P. agglomerans ISIB55-polymer mix and (B) B. caribensis ISIB40-polymer mix. The micrographs were observed at 5000x magnification. Results are representative of three independent experiments. (TIF) [file pone.0176930.s002.TIF]
